# Thin Film Nanocomposite Membrane Filled with Metal-Organic Frameworks UiO-66 and MIL-125 Nanoparticles for Water Desalination

**DOI:** 10.3390/membranes7020031

**Published:** 2017-06-14

**Authors:** Mohammed Kadhom, Weiming Hu, Baolin Deng

**Affiliations:** 1 Department of Chemical Engineering, University of Missouri, Columbia, MO 65211, USA; makbq6@mail.missouri.edu; 2 Al-Dour Technical Institute, Northern Technical University, Al-Dour, Saladin, Iraq; 3 Department of Civil and Environmental Engineering, University of Missouri, Columbia, MO 65211, USA; why78@mail.missouri.edu

**Keywords:** metal-organic framework, thin film nanocomposite membrane, reverse osmosis, desalination, nanoparticles

## Abstract

Knowing that the world is facing a shortage of fresh water, desalination, in its different forms including reverse osmosis, represents a practical approach to produce potable water from a saline source. In this report, two kinds of Metal-Organic Frameworks (MOFs) nanoparticles (NPs), UiO-66 (~100 nm) and MIL-125 (~100 nm), were embedded separately into thin-film composite membranes in different weight ratios, 0%, 0.05%, 0.1%, 0.15%, 0.2%, and 0.3%. The membranes were synthesized by the interfacial polymerization (IP) of *m*-phenylenediamine (MPD) in aqueous solution and trimesoyl chloride (TMC) in an organic phase. The as-prepared membranes were characterized by scanning electron microscopy (SEM), transmission electron microscopy (TEM), contact angle measurement, attenuated total reflection Fourier transform infrared (ATR FT-IR) spectroscopy, and salt rejection and water flux assessments. Results showed that both UiO-66 and MIL-125 could improve the membranes’ performance and the impacts depended on the NPs loading. At the optimum NPs loadings, 0.15% for UiO-66 and 0.3% for MIL-125, the water flux increased from 62.5 L/m^2^ h to 74.9 and 85.0 L/m^2^ h, respectively. NaCl rejection was not significantly affected (UiO-66) or slightly improved (MIL-125) by embedding these NPs, always at >98.5% as tested at 2000 ppm salt concentration and 300 psi transmembrane pressure. The results from this study demonstrate that it is promising to apply MOFs NPs to enhance the TFC membrane performance for desalination.

## 1. Introduction

Reverse osmosis (RO) is now the most employed method of desalination to convert saline or brackish water source to freshwater. The process uses a membrane as a selective barrier through which water molecules, but not the salts or organics, pass under pressure [1]. Desalination by various approaches, especially RO, represents a solution to address the water shortage problem that has become increasingly serious in the recent decades [2]. Development of RO processes and membranes has gone through many stages and forms since the first applicable membrane synthesis in the early 1960s [3]. The state-of-the-art membrane is the thin film composite (TFC) membrane invented by John Cadotte in the early 1980s. The TFC membrane has a polyamide dense layer with a thickness of a few hundred nanometers supported by an ultrafiltration membrane. This polyamide layer is the active layer for salt rejection, formed by the interfacial polymerization of the MPD dissolved in water and TMC dissolved in organic solvent [4].

Numerous researches have been devoted to developing and improving the performance of TFC membranes [5]. One approach is to embed nano-sized materials such as zeolite [6,7,8,9,10,11,12], silica [13,14,15,16,17,18], titanium dioxide [19,20,21,22], or carbon nanotubes [23,24,25] inside the membrane to improve its properties and performance. Another way is to select proper materials and control the manufacturing conditions affecting the membrane’s efficiency [6,26,27,28,29]. In addition, the engineering process could be optimized to increase the pure water production efficiency and lower the energy cost [30,31,32,33,34].

Metal-Organic Frameworks (MOFs) are a class of materials consisting of an inorganic or metal core surrounded by an organic linker material [35]. MOFs have very high specific surface area, a high number of adsorption sites, different particle structures, distinct pore size and structure, and can be applied for various purposes [35,36,37,38,39]. These include applications for ion exchange [40], light harvesting [41], sensing [42], catalyst [43], drug delivery [44], gas storage [45,46], and separation [47,48]. Such a wide range of applications are possible because MOFs can be easily designed toward desired uses.

MOFs have been widely used for gas separation. Their porous structure can generate exceptionally high pore volume and surface area reaching over 7000 m^2^/g [49], which is particularly suitable for gas storage and separation. Beginning with the first MOFs membrane synthesized and used for gas separation [50], different forms of MOFs membranes have been prepared, often on an organic or inorganic support layer. MOFs particles have been incorporated into polyamide layer of thin film nanocomposite (TFN) membrane with improved properties [35,51]. In water treatment, MOFs have been used as adsorbents of heavy metals and as fillers in alumina hollow fiber membranes for water desalination [52,53]. MOFs have also been employed in TFN membrane to treat water in different applications. Lee et al. used A100 (aluminum terephthalate) and C300 (copper benzene-1,3,5-tricarboxylate) as water soluble MOFs, which were embedded and dissolved into the ultrafiltration membrane to increase its porosity [54]. For reverse osmosis, Hu et al. [55] and Gupta et al. [56] reported two simulation studies that used Zeolitic Imidazolate Framework (ZIFs)—MOFs based on zeolite as membranes for water desalination. Xu et al. [57] filled MIL-101(Cr) NPs into the TFN membrane to improve the desalination performance. Findings showed that the water flux increased by 44% comparing to the plain membrane by adding 0.05% MIL-101 NPs to the organic solution, while the salt rejection remained almost the same. Similar outcomes were observed by Ma et al. [58] when embedding UiO-66 in membranes used in forward osmosis. By loading 0.1% NPs to the membrane, the water flux increased from 2.19 to 3.33 LMH/bar. Nevertheless, the use of MOFs membranes in water treatment is still in its infancy when compared with their applications for gas separation.

**Figure 1:**
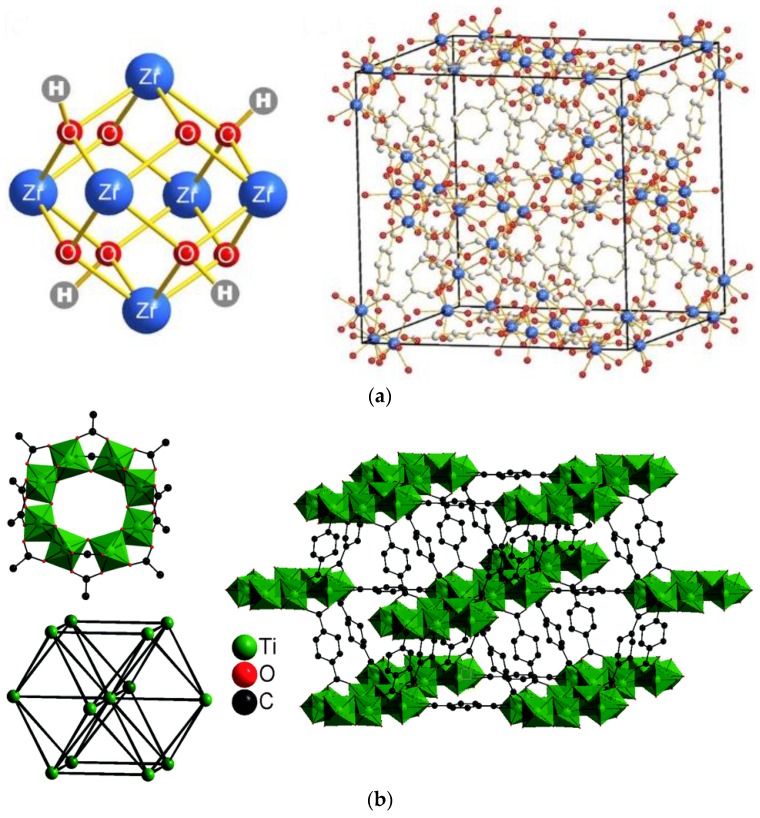
Metal-Organic Frameworks (MOFs) structure: (**a**) UiO66 [52], reproduced with permission from Springer Nature and (**b**) MIL-125 [62] reproduced with permission from Royal Society of Chemistry.

UiO-66 (MOFs based on zirconium) [59] and MIL-125 (MOFs based on titanium) [60] are water stable, carboxylate ligand–based MOFs [61]. The materials possess many metal atom centers, which give them high structural symmetry and steadiness. Figure 1 shows these structures as reported by Wang et al. [52] for UiO-66 and Devic and Serre for MIL-125 [62]. Properties including their high surface area, chemicals resistance, and functionalization ability made these MOFs good candidates for different applications [63,64].

In this paper, UiO-66 and MIL-125 MOFs nanoparticles were synthesized and then embedded into the TFN membrane. The NPs were incorporated by mixing different amounts of materials (0–0.3 wt %) in the trimesoyl chloride solution during membrane fabrication, and the membranes’ performance in reverse osmosis application was tested. MOFs NPs were characterized by SEM, zeta potential measurement, and surface area evaluation, while the membranes’ physicochemical properties were examined by SEM, TEM, contact angle, and ATR FT-IR tests. It was found that embedding UiO-66 and MIL-125 NPs to the TFN membrane improved its performance to a level that is considered to be among the best for brackish water desalination.

## 2. Materials and Methods

### 2.1. Materials

Zirconium (IV) chloride (ZrCl_4_, 99.5%), titanium (IV) isopropoxide (Ti [OCH(CH_3_)_2_]_4_, 97%), 1,4-benzenedicarboxylic acid (BDC, 98%), dimethylformamide (DMF, 99.9%), and methanol (CH_3_OH, 99.8%) were obtained from Sigma-Aldrich (St. Louis, MO, USA) for the preparation of MOFs NPs.

The PSU support sheets were prepared by dissolving polysulfone pellets (PSU, *M*_W_ = 35,000, Sigma-Aldrich) in DMF solvent. The chemicals for the IP, *m*-phenylenediamine (MPD, ≥99%) and trimesoyl chloride (TMC, ≥98.5%), were purchased from Fisher Scientific (Pittsburgh, PA, USA) and Sigma Aldrich, respectively. 2,2,4-trimethylpentane (isooctane, 99%) was obtained from Fisher Scientific and used as TMC solvent. Our recent study demonstrated that isooctane could replace commonly used hexane during the IP process as a more environmental-friendly solvent [18]. Millipore DI water (Synergy185, 18.2 MΩ-cm, EMD Millipore Corp., Billerica, MA, USA) was used to prepare MPD solution and for cleaning purposes. Calcium chloride (CaCl_2_) and sodium chloride (NaCl) were obtained from Fisher Scientific and Sigma Aldrich, respectively. CSA/TEA salt materials, triethylamine (TEA, ≥99%) and (1s)-(+)-10-camphorsulfonic acid (CSA, 99%), were purchased from Sigma Aldrich.

### 2.2. Synthesis and Characterizations of MOFs NPs

UiO-66 and MIL-125 MOFs NPs used in this research were synthesized through the microwave and solvothermal methods, respectively. For UiO-66 preparation, ZrCl_4_, BDC, and H_2_O were dissolved in DMF under stirring in molar ratios of 1:1:1:500, respectively [52]. After a complete mixing, the solution was placed in a GE microwave (Model no. J VM131K 002) and irradiated for 5 min at a power of 220 W. The 5 min irradiation was separated into 4 steps: 2 min, 1 min, 1 min, and 1 min with 1 min relaxing time between each irradiation section to prevent solvent from heating over boiling point. The MOF precipitates appeared during the last 2 steps of irradiation. MIL-125 NPs were prepared in different formula, in which 3 mmol Ti[OCH(CH_3_)_2_]_4_, 6 mmol BDC, 50 mL methanol, and 20 mL acetic acid were dissolved in 100 mL DMF [60]. After mixing, the solution was transferred to a 500 mL Teflon liner and put into a metallic digestion bomb at 150 °C for 24 h. Precipitates of both MOFs were washed and centrifuged with DMF and methanol, three times each. The washed samples were dried in an oven overnight at 80 °C and collected for later use.

The NPs size and morphology were tested by SEM (Hitachi S-4700 Field Emission Scanning Electron Microscope, Hitachi, Ltd., Tokyo, Japan). First, the particles were spread on a carbon adhesive disc. After that, they were coated with platinum on a sputter coater (K575x, Emitech, Kent, UK) for 1 min at 20 milliAmps, prior to imaging. Zetasizer (Malvern nano series, Malvern Instruments Ltd, Malvern, UK) was used to measure the NPs zeta potential. 0.1 g of the NPs was placed in 20 mL DI water of pH 5.7 and sonicated for 10 min to avoid aggregation. The specific surface area was determined by N_2_-adsorption using the Beckman coulter SA 3100 (Beckman Coulter, Inc., Brea, CA, USA) surface area analyzer, according to the Brunauer-Emmett-Teller (BET) method.

### 2.3. Preparation of PSU Support Sheets

The casting solution was made by dissolving 15 wt % of PSU grains in DMF. The solution was heated to 60 °C and stirred for at least 5 h, till a colorless solution formed. The solution was removed from the heater and cooled down at room temperature while allowing vent of the organic vapor; then left overnight for degassing.

A solution aliquot was spread over a glass plate and cast to a thickness of 130 µm by a casting knife (EQ-Se-KTQ-150, MTI Corp., Richmond, CA, USA). The glass plate with the PSU solution was directly immersed into a water bath. The colorless solution then turned to a white sheet instantly and detached from the glass in a few seconds, in a process of phase inversion. The support sheets were collected and washed three times in DI water for the removal of any remaining solvent. Finally, the sheets were stored in DI water at 4 °C for at least 24 h before use.

### 2.4. Preparation of TFN Membranes

The prepared support sheet was placed on a piece of glass with the excess water removed by a squeegee roller. MPD solution was then poured on the PSU sheet for 25 s, then excess solution was removed by the squeegee roller. MPD solution was prepared by dissolving 2 wt % of MPD in DI water. The solution also contained 1% of CAS/TEA salt and 0.01% CaCl_2_, which were added to improve membrane’s hydrophilicity and performance. The membrane with MPD was left for 2 min to dry, then the TMC solution, which was prepared by dissolving 0.15 wt % TMC in isooctane, was added to the sheet for 15 s, and the reaction of TMC and MPD led to the formation of a thin polyamide film by the interfacial polymerization (IP). After disposing the extra TMC solution, the sheets were dried in oven at 80 °C for 6 min for the evaporation of any residual solvent. Finally, the sheets were washed in DI water three times and stored in water at 4 °C for at least 18 h before test. Since the MOFs have higher dispersion affinity in organic solutions than in water due to the relatively hydrophobic organic shells, the MOFs NPs were dispersed in TMC solution in different loading ratios, 0.05, 0.1, 0.15, 0.2, and 0.3 wt %.

### 2.5. TFN Membrane Characterizations

The prepared membranes were dried at room temperature for around 24 h and stored at 4 °C prior to characterizations. The membrane’s morphology was examined by SEM (Hitachi S-4700). The samples were coated with platinum by a sputter coater (Emitech, K575x) for 1 min at 20 milliAmps. After placing the samples inside the SEM device, different voltages were used to achieve various resolutions.

The membrane’s cross sectional view was observed using TEM (JEOL JEM-1400, JEOL Ltd., Peabody, MA, USA) device. The samples were prepared by soaking them in a resin (Eponate 12, Ted Pella, Inc., Redding, CA, USA) overnight, then cut by the Reichert–Jung Ultracut E ultramicrotome (Reichert, Depew, NY, USA).

The membrane surface functional groups were assessed by the ATR FT-IR spectroscopy, using the Nicolet 4700 FT-IR with the multi-reflection Smart Performer^®^ ATR accessory (Thermo Electron, Waltham, MA, USA).

The contact angle between DI water drop and membrane’s surface was measured to understand the surface hydrophilicity. A sessile drop technique-based contact angle video system, VCA-2500 XE (AST products, Inc., Billerica, MA, USA), was employed for this measurement. Each reported value of contact angle was the average of six measurements tested on different locations.

A cross flow reverse osmosis system was used to measure the water flux and salt rejection as shown in our previous work [18]. The membrane was placed in a filter holder cell (HP Filter Holder, 47 mm, stainless steel, EMD Millipore, Billerica, MA, USA) and tested, under conditions of 300 psi, 25 °C, and 2000 ppm NaCl solution, for eight hours. The permeate water flux was calculated based on its transferred volume per unit time; the LABVIEW software was used to track the water flux, calculated based on Equation (1).

(1) $$J=\frac{V}{A\times \;t}$$

where *J* is the water flux (L/m^2^ h), *V* the permeate water volume (L), *A* the membrane area (m^2^), and *t* the accumulation time (h).

A conductivity meter (HACH Company, Loveland, CO, USA) was employed to measure the total dissolved salts in feed solution and permeate water. NaCl rejection was calculated by Equation (2).

(2) $$R=\left(1-\frac{C_{p}}{C_{f}}\right)\times 100$$

where *R* is the salt rejection ratio, *C_p_* the permeate conductivity and *C_f_* the feed conductivity.

The salt solubility maintained the same by controlling the system temperature at 25 °C using external water bath.

## 3. Results and Discussion

### 3.1. MOFs NPs Characterizations

UiO-66 and MIL-125 NPs had measured surface areas of 293.6 and 515.5 m^2^/g, respectively. Although these numbers seem to indicate the materials are highly porous, they are low compared with what had been reported in the literature [60,65]. The reason for this difference is not clear but the materials’ aggregation could have contributed to the difference.

The zeta potential values of UiO-66 and MIL-125 NPs were 7.66 mV and −46.1 mV, respectively, when they were tested at a concentration of 0.1% in DI water. Although both zirconium and titanium could be in the oxidation state of +4, different surface charges, positive and negative, were measured for UiO-66 and MIL-125, respectively. The measured zeta potential of UiO-66 at pH 5.7 was close to the literature-reported value of 0 mV at almost the same pH 5.5 [66]. MIL-125’s zeta potential was lower than what was previously reported, around +2 mV at pH of 5.5 [67,68]. This could be due to the use of different titanium raw materials; titanium isopropoxide was used in this research instead of titanium butoxide used in previous studies.

**Figure 2:**
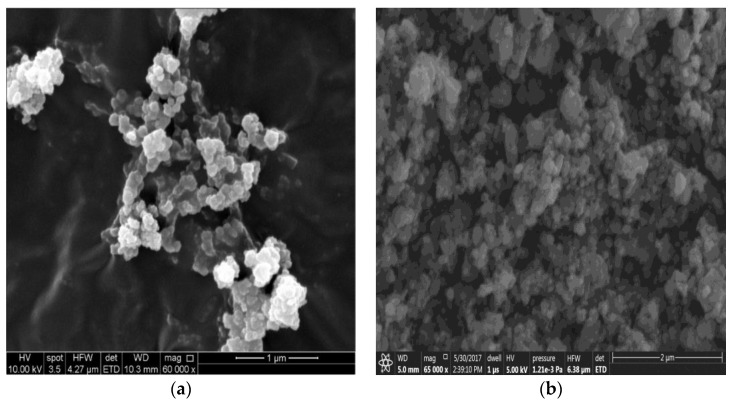
Scanning electron microscopy (SEM) images for (**a**) UiO-66 and (**b**) MIL-125 MOFs nanoparticles (NPs).

Figure 2a,b presents the SEM morphology of UiO-66 (100–200 nm, cubic shape) and MIL-125 (100 nm, spherical shape) respectively. The sizes of NPs were appropriate to be embedded in TFN membranes, as it would be shown later.

### 3.2. TFN Membrane Characterizations

**Figure 3:**
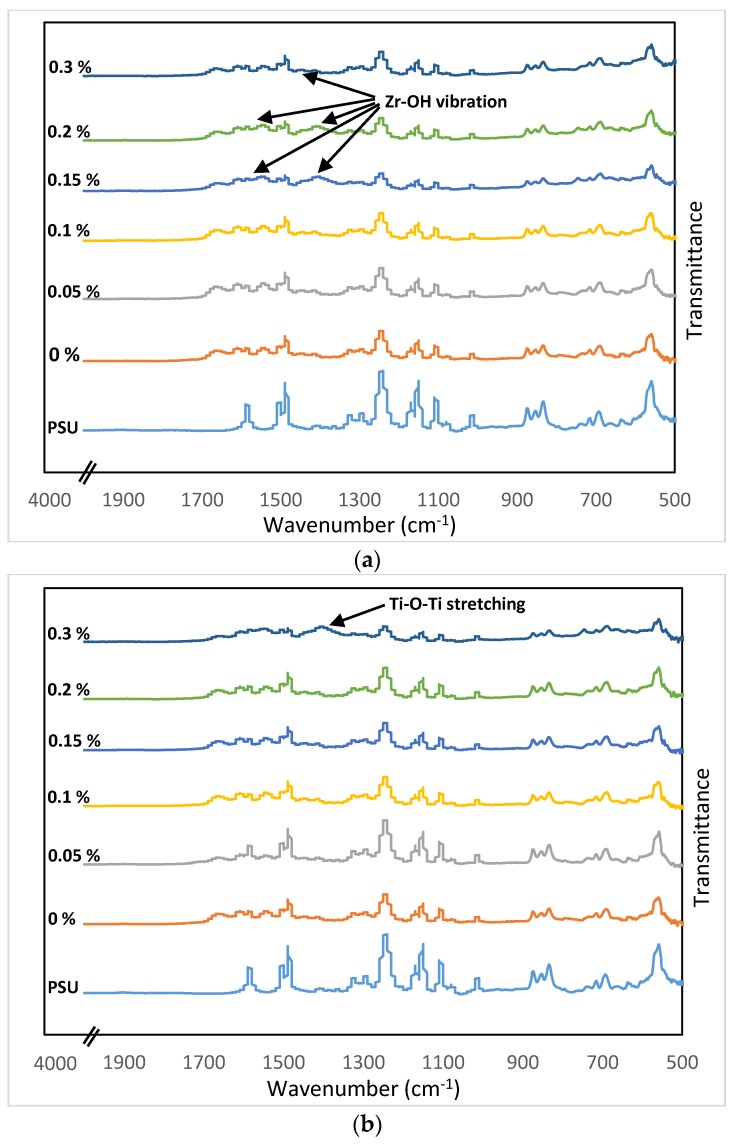
Attenuated total reflection Fourier transform infrared (ATR FT-IR) spectra for (**a**) UiO-66 and (**b**) MIL-125.

ATR FT-IR spectra were collected from the surface of various membrane specimens. The spectra of the PSU support sheet, TFC membrane, and the TFN membranes with UiO-66 and MIL-125 NPs, respectively, were illustrated in Figure 3a,b. The spectral features of the PSU layer and TFC membrane chemical groups are repeated in TFN membranes spectra, in which the peaks corresponding to MOFs were also observed. Generally, by increasing NPs percentage, the reflected spectra had new peaks relevant to the embedded materials. Starting with the PSU spectrum, two featured peaks at 1150 and 1245 cm^−1^ were observed, which could be allocated to the O=S=O symmetric stretching of sulfone group [69] and C–O–C asymmetric stretching of aryl ethyl group, respectively [70]. The peaks at 1298 and 1325 cm^−1^ could indicate the asymmetric stretching of O=S=O sulfone group. At 1488 and 1590 cm^−1^, the peaks could be attributed to the aromatic C–C stretching [69,70]. By adding polyamide thin film layer, peaks at 1350 and 1610 cm^−1^ appeared due to the N–H deforming (amide III) and C=O (carboxylic), respectively [71]. Furthermore, N–H bending and C–N stretching could be represented by the peak around 1545 cm^−1^ (amide II). The vibration at 1660 cm^−1^ could be assigned to the C=O stretching (amide I) [71,72].

When MOFs NPs were added at very low amounts, new peaks corresponding to these NPs were not apparent. By increasing UiO-66 molar ratio to 0.15 and above, an obvious peak around 1380 cm^−1^ and a tiny one at 1565 cm^−1^ appeared, which could be assigned to the Zr-OH vibration [73,74,75,76], as shown in Figure 3a. At 0.3%, the peak disappeared; this might be caused by the particles’ aggregation so no proper NP dispersion was obtained. For MIL-125 at 0.3%, a clear peak was observed around 1416 cm^−1^, which could be assigned to the Ti–O–Ti stretching vibrations [77].

**Figure 4:**
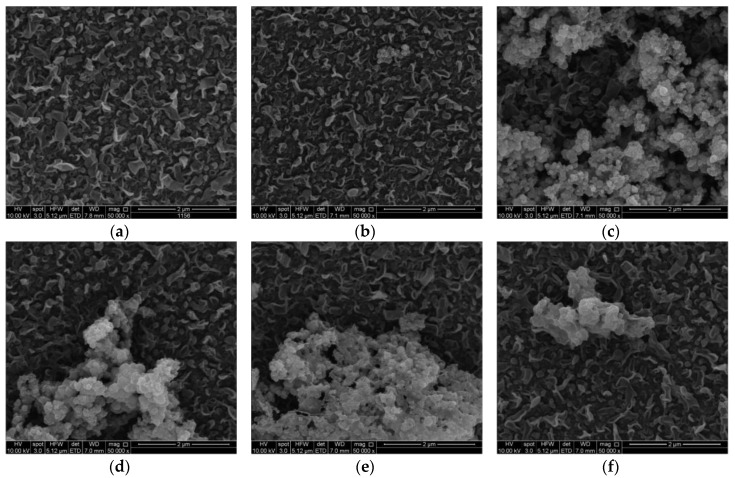
SEM images for membranes surface with UiO-66 NPs injection, (**a**) 0%, (**b**) 0.05%, (**c**) 0.1%, (**d**) 0.15%, (**e**) 0.2%, and (**f**) 0.3%.

**Figure 5:**
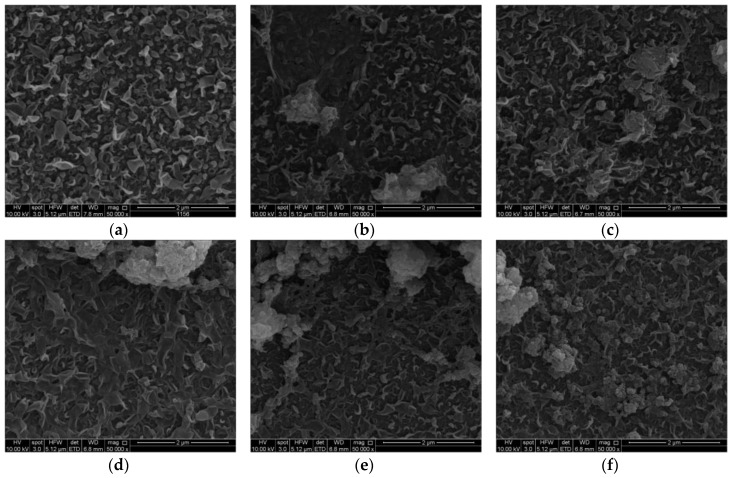
SEM images for membranes surface with MIL-125 NPs injection, (**a**) 0%, (**b**) 0.05%, (**c**) 0.1%, (**d**) 0.15%, (**e**) 0.2%, and (**f**) 0.3%.

The SEM images of membranes surface with UiO-66 and MIL-125 NPs are illustrated in Figure 4 and Figure 5, respectively. The TFC membrane, for which there was no particles added, showed a leaf-like shape characteristic of normal polyamide layer. Addition of up to 0.05% UiO-66 NPs did not cause much change in leaf-like morphology, as illustrated in Figure 4b. When the loading was increased to 0.1% and 0.15%, the membranes’ surface began to change due to NPs filling into the membrane. The nanoparticles covered a wide area and had a good distribution in the membrane structure. At 0.2% and 0.3%, the solid clusters/aggregates were easily observable, which might have an impact on salt rejection. At loading of 0.3%, the NPs aggregated and formed even larger clusters, and covered the surface more sparsely. This is consistent with the FT-IR results and trends, as discussed in the previous section.

In Figure 5, the impact of embedding MIL-125 NPs on the morphology was presented. Unlike UiO-66 filling, MIL-125 appeared to have changed the leaf-shaped morphology, in addition to the aggregation. The leaves are connected to each other, making long leaves. This could be because of MIL-125’s quasi-cubic tetragonal structure, or the smaller size of MIL-125 comparing with UiO-66 NPs, which might give better filling into the membrane.

**Figure 6:**
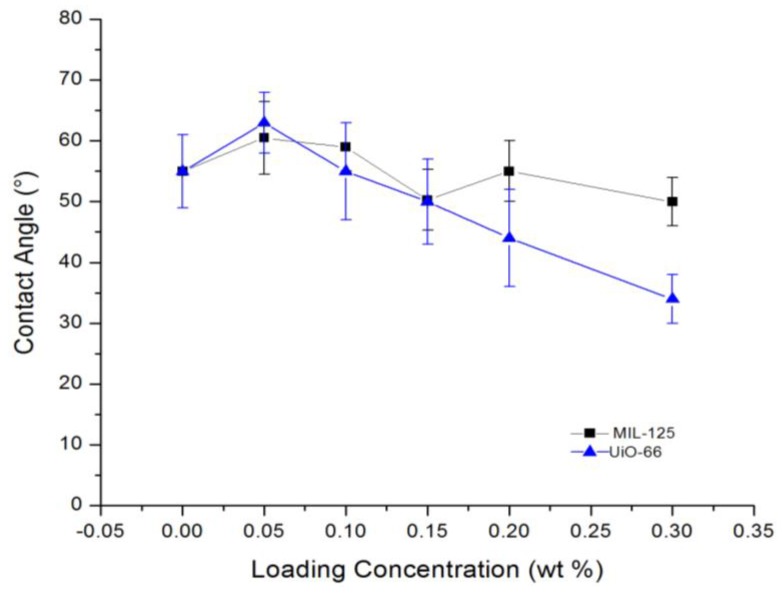
Pure water contact angle with the modified thin film nanocomposite (TFN) membranes surface.

Figure 6 shows the measured contact angles between DI water drop and the membrane’s surface. By adding the MOFs NPs, the contact angle did not decrease significantly. The result is different from filling other nanomaterials such as hydrophilic silica [18] and oxidized graphene [78] where significant decreases of contact angle were observed. This may be attributed to the MOFs structure, which contains organic linkers surrounding the metal core. The organic part is slightly hydrophobic, leading to decreased particle hydrophilicity [38]. From the figure, the UiO-66 had more hydrophilic effect than MIL-125. This could be because MIL-125 has more linkers on the surface shielding the metal atom. The observation is also in consistence with the fact that MIL-125 had a higher surface area.

**Figure 7:**
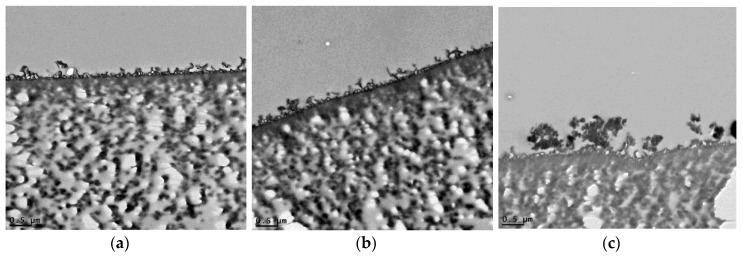
Transmission electron microscopy (TEM) images of (**a**) thin film composite (TFC) membrane, (**b**) TFN membrane of 0.15% UiO-66, and (**c**) TFN membrane of 0.3% MIL-125.

TEM cross section images for the plain membrane and UiO-66 and MIL-125 nanocomposite membranes are illustrated in Figure 7a–c, respectively. Figure 7b is the cross-sectional view of TFN membrane at an optimal UiO-66 NPs loading (0.15%); while Figure 7c is at an optimal MIL-125 NPs loading (0.3%). The TEM examination showed that the particles were filled into the membranes and appeared as black clusters; MIL-125 NPs were highly homogenized with the membrane and affected its texture. From the images, the membrane thickness is found to be 200–400 nm.

### 3.3. TFN Membranes Performance

**Figure 8:**
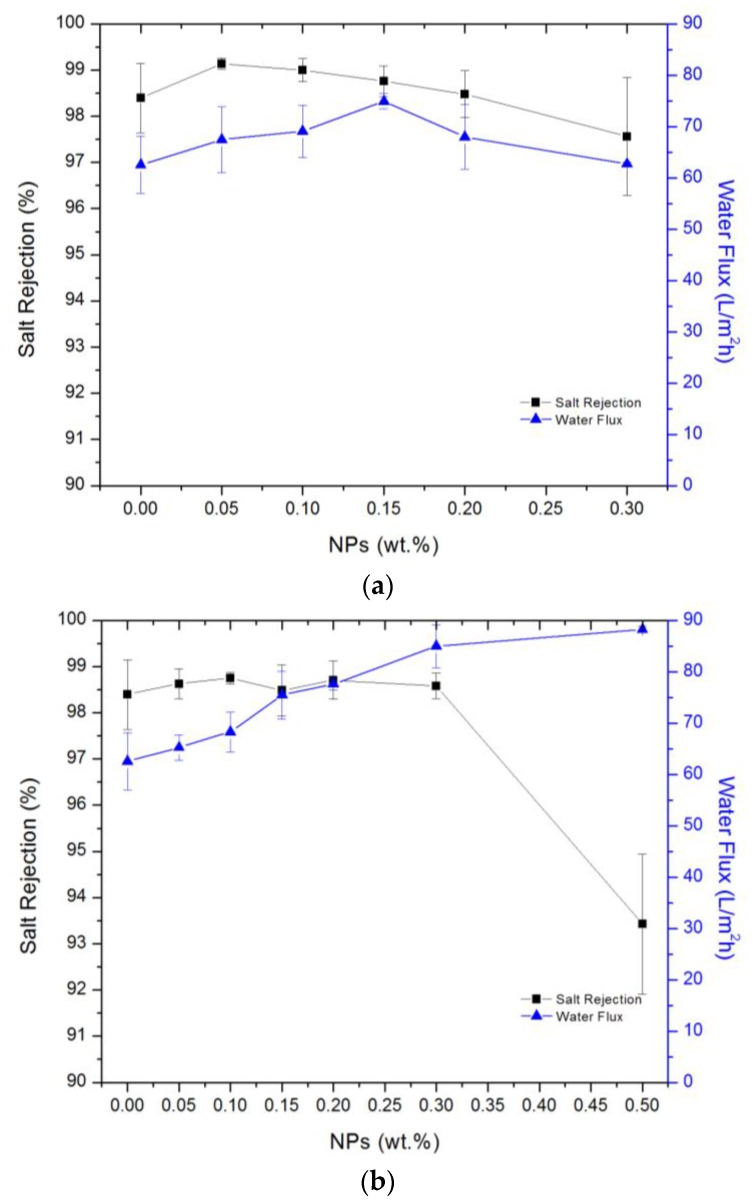
TFN membranes performance with injected (**a**) UiO-66 and (**b**) MIL-125 NPs.

The salt rejection and water flux for UiO-66 and MIL-125 modified membranes were documented in Figure 8a,b. For UiO-66, the optimal addition was 0.15%, where the permeate water flux and salt rejection increased from 62.5 L/m^2^ h and 98.4% to 74.9 L/m^2^ h and 98.8%, respectively. From Figure 8a, adding UiO-66 NPs increased NaCl rejection at low loadings, but a further increase of the NPs resulted in a decrease of salt rejection. The rejection increase at low loading could be attributed to UiO-66’s pore size (∼6.0 Å) [59], which is larger than water molecule (~2.8 Å) but smaller than the hydrated ions (Na^+^ = 7.16 and Cl^−^ = 6.64 Å) [79]. Increasing the NPs amount too much could decrease salt rejection, as the particles aggregation may generate cracks in TFN membranes structure. On the other hand, the flux increase by adding NPs reached its maximum at 0.15%. This is likely due to the hydrophilic nature of UiO-66 [59,80]. At percentages higher than 0.15%, the water flux decreased, again likely due to NPs aggregation [13]. Here, both salt rejection and water flux decreased because of particle aggregation. Aggregation may have created micro gaps between particle blocs that allowed the saline water to pass through. The particle aggregates could also block the water transfer through the membrane by the hydrophobic linkers overlapping, thus decreased water flux. Thereby, reduction in both salt rejection and water flux occurred.

**Figure 9:**
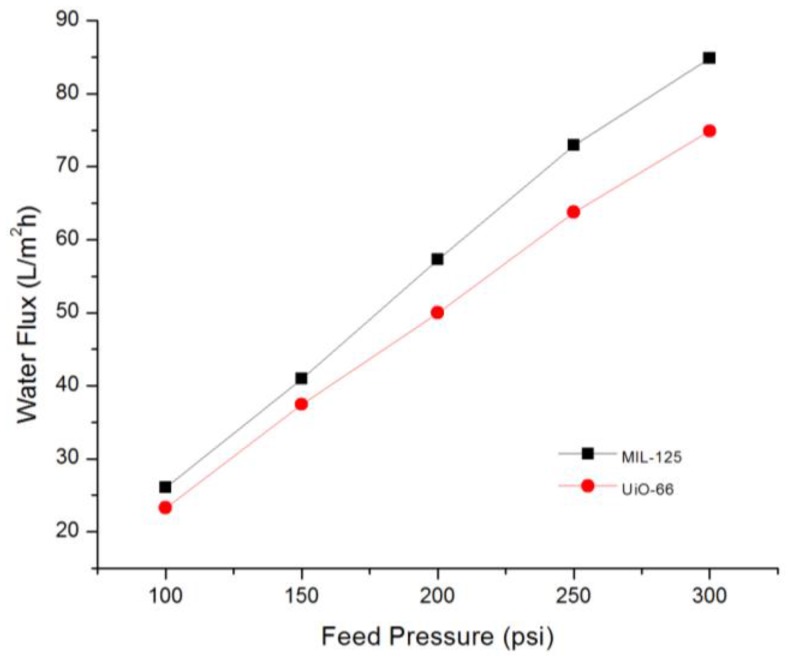
Membranes permeability at an optimal NPs filling.

Figure 8b shows the effect of adding MIL-125 NPs to TFN membrane. The water flux increased by increasing NPs loading, for example, with a solid loading of 0.3%, the permeate water flux was increased from 62.5 to 85.0 L/m^2^ h, while the salt rejection maintained almost the same (98.4% vs. 98.6%). At filling of 0.5% NPs, a drop in salt rejection happened. The effective pore diameter of MIL-125 is 12.55 Å [60] which is larger than both the water molecule and hydrated ions of Na^+^ and Cl^−^. However, the salt rejection remained the same even at a higher loading, the high negative charge of MIL-125 could play a role here as the formed electrical double layer could repel the negatively-charged ions. The reported water flux here at 0.3%, 85.0 L/m^2^ h, is considered one of the highest in comparison with the reported values in the literature [81]. The high flux could be due to the NPs hydrophilicity and their large surface area and pore size. Figure 9 shows the permeability of optimal membranes by adding 0.15 wt % of UiO-66 or 0.3 wt % MIL-125 at different feed pressures.

### 3.4. Why MOFs for TFN Membranes?

**Figure 10:**
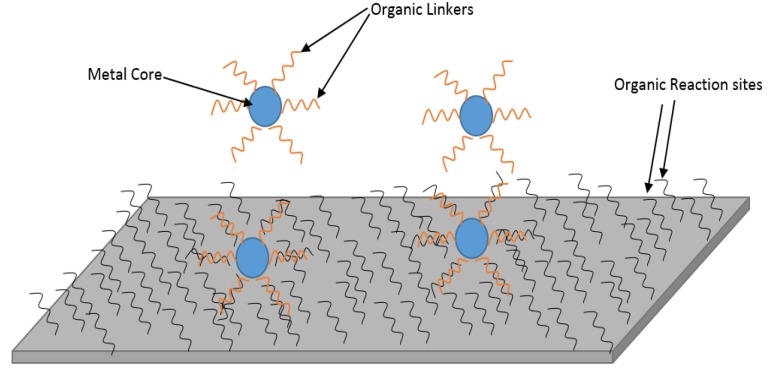
MOFs filling into polymeric substance diagram.

MOFs are valuable materials due to their unique structure and wide applications in various areas [35]. It is anticipated that by the coming decade, mixed matrix membranes based on MOFs could grow, including those for large scale applications [38]. Among various nanocomposite membranes, MOFs-enabled membranes are advantageous because they can overcome the traditional filling problems of low affinity with the membrane, porosity blocking, and segregation [82,83]. The hybrid MOFs structure with organic and inorganic parts is generally compatible with polymeric layers in membrane [84]. This concept is illustrated in Figure 10, where MOFs particles can be more conveniently embedded into the TFN membrane with less gaps than those created by traditional inorganic fillers. Another advantage is the low cost of the polymeric membranes and the vast selection range of linkers toward controlling MOFs shape, morphology, and surface chemistry, making it potentially possible to design membrane for specific applications.

## 4. Conclusions

Incorporation of UiO-66 and MIL-125 MOFs NPs in reverse osmosis membranes is reported in this study. The results showed that MOFs were embedded well inside the membranes and increased the water flux and salt rejection. Different techniques were used to exam the NPs and TFN membranes’ physicochemical properties. The organic linker enhanced the compatibility of MOFs particles with the polyamide thin film. The results indicated that the water flux was increased from 62.5 to 74.9 or 85.0 L/m^2^ h at a transmembrane pressure of 300 psi, by addition of 0.15% of UiO-66 or 0.3% of MIL-125, respectively, while salt rejection was slightly increased. These high flux results indicate filling of MOFs NPs in TFN membrane might be advantageous because of the NPs containing an organic part that could link properly to polyamide, and inorganic part that could enhance the membrane performance.
